# Cyclin D1 cooperates with p21 to regulate TGFβ-mediated breast cancer cell migration and tumor local invasion

**DOI:** 10.1186/bcr3441

**Published:** 2013-06-20

**Authors:** Meiou Dai, Amal A Al-Odaini, Nadège Fils-Aimé, Manuel A Villatoro, Jimin Guo, Ani Arakelian, Shafaat A Rabbani, Suhad Ali, Jean Jacques Lebrun

**Affiliations:** 1 Division of Medical Oncology, Department of Medicine, McGill University Health Center, Royal Victoria Hospital, Montreal, QC, Canada; 2 University of Dammam, Ministry of Higher Education, Saudi Arabia; 3 Department of Medicine, McGill University Health Center, Royal Victoria Hospital, Montreal, QC, Canada

**Keywords:** Cyclin D1, p21Cip1, Transforming growth factor beta (TGFβ), Breast cancer, Migration, Invasion

## Abstract

**Introduction:**

Deregulation of the cell cycle machinery is often found in human cancers. Modulations in the cell cycle regulator function and expression result not only in proliferative advantages, but also lead to tumor progression and invasiveness of the cancer. In particular, cyclin D1 and p21 are often over-expressed in human cancers, correlating with high tumor grade, poor prognosis and increased metastasis. This prompted us to investigate the role of the cyclin D1/p21 signaling axis downstream of transforming growth factor beta (TGFβ) in breast cancer progression.

**Methods:**

Cyclins mRNA and protein expressions were assessed by quantitative real-time PCR and Western blot in triple negative breast cancer cell lines. Co-localization and interaction between cyclin D1 and p21 were performed by immunocytochemistry and co-immunoprecipitation, respectively. Cell migration was assessed by wound healing and quantitative time-lapse imaging assays. In addition, the effects of cyclin D1 on cellular structure and actin organization were examined by staining with F-actin marker phalloidin and mesenchymal intermediate filament vimentin. Finally, a mammary fat pad xenograft mouse model was used to assess mammary tumor growth and local invasion.

**Results:**

We found TGFβ to specifically up-regulate the expression of cyclin D1 in triple negative breast cancer cells. Induction of cyclin D1 is also required for TGFβ-mediated cell migration. Suppression of cyclin D1 expression not only resulted in a rounded and epithelial-like phenotype, but also prevented TGFβ-induced vimentin and F-actin co-localization at the cell edge as well as invadopodia formation. Furthermore, TGFβ promoted the nuclear co-localization and physical interaction between cyclin D1 and p21. The co-expression of cyclin D1 and p21 proteins are required for the initial steps of tumor development, as double knockdown of these two molecules prevented primary tumor formation in a Xenograft mouse model. Moreover, the *in vivo *studies indicated that locally advanced features of the invasive tumors, including skeletal muscle, mammary fat pad and lymphovascular invasion, as well as ulcerated skin, were attenuated in the absence of cyclin D1 and p21.

**Conclusions:**

Thus, our findings highlight the cyclin D1/p21 signaling axis as a critical regulator of TGFβ-mediated tumor growth initiation and local tumor cell invasion, both *in vitro *and *in vivo*.

## Introduction

Metastatic cancer is a largely incurable disease and responsible for 90% of human cancer deaths [1]. To develop metastasis in a distant organ, cancer cells must initially disseminate from the primary tumor and invade through the surrounding basement membrane and stroma into lymphatic or blood vessels, followed by survival, extravasation and re-implantation at a secondary site [2]. As cancer cell motility and invasiveness are critical features in the initial development of metastasis, many molecules involved in these processes are becoming attractive therapeutic targets [3]. Understanding the molecular mechanisms that govern these early processes may provide insightful strategies for the prevention of cancer progression and metastasis.

The transforming growth factor beta (TGFβ) superfamily is comprised of many members, including activins, anti-Müllerian hormone, bone morphogenetic proteins, growth and differentiation factors, inhibins and TGFβs [4]. Among these family members, TGFβ ligands and its receptors are widely expressed in all tissues and the regulatory role played by these growth factors is of central importance to human cancer development and progression. TGFβ can be released from storage sites in the extracellular matix (ECM) and bone, as well as secreted in a paracrine and autocrine manner by platelet, myeloid, mesenchymal and cancer cells [5-7]. The increasing amount of TGFβ1 is correlated with a high incidence of distant metastasis as TGFβ acts on the tumor cells and the surrounding stroma to promote epithelial to mesenchymal transition (EMT), ECM degradation, cell migration, cell invasion, angiogenesis, immunosuppression and modification of the tumor microenvironment [8-11]. Intravital imaging of live tumor-bearing nude mice demonstrated that active TGFβ signaling is heterogeneously distributed in a minority of cancer cells within primary mammary tumors [12]. The activation of TGFβ signaling promotes single tumor cell migration and metastatic spread into blood vessels and lymph nodes. However, not all cells with active TGFβ signaling are migratory, suggesting differential TGFβ signaling events and specific downstream targets are required for this process.

TGFβ signal transduction begins with ligand binding to the TGFβ type II receptor, which recruits and activates the type I receptor. The activated type I receptor then phosphorylates intracellular mediators known as receptor-regulated Smads (R-Smads), Smad2 and Smad3. This phosphorylation event allows for subsequent heterotrimerization of two phosphorylated R-Smad subunits with one common partner, Smad4 [13,14]. The Smad heterotrimer then translocates to the nucleus where it can bind DNA, but with a very low affinity [15]. In order to achieve high affinity binding, the Smads associate with various DNA binding partners [16]. It is thought that these partner proteins, which act as co-activators or co-repressors, are functionally expressed in different cell types, thus providing a basis for tissue and cell type-specific functions for TGFβ ligands [17].

Perturbations in the regulation of the cell cycle machinery often occur in human cancers, resulting in an imbalance between cell growth and cell death [18]. In addition, several reports have proposed that deregulation of cell cycle regulators results not only in proliferative advantages, but also in increased tumor progression and aggressiveness traits [19]. Cell cycle progression is primarily mediated through interactions between the different cyclins with their respective cyclin-dependent kinases (CDKs). Among the different cyclins, cyclin D1 and cyclin E are associated with the G1-S phase transition [20]. Cyclin D1 interacts with CDK4 and 6, while cyclin E interacts more specifically with CDK2 [21-25]. The activity of the cyclin-CDK complexes is regulated by two classes of small proteins referred to as cyclin-dependent kinases inhibitors (CDKIs). The INK4 family, which includes p15INK4, p16INK4A, p18INK4C and p19INK4D, specifically binds to CDK4 and 6, thereby preventing their association with the D-type cyclins [26-29]. The KIP family includes p21CIP1/WAF1 (p21), p27KIP1 and p57KIP2 [30-35]. While the KIP family members are usually associated with cyclin E-CDK and cyclin A-CDK complexes, many reports indicated that they also interact with cyclin D-CDK complexes [30,36-38].

Many of these cell cycle regulators are primary targets of TGFβ signaling in human cancers [39-41]. Interestingly, some of these cell cycle regulators, in particular cyclin D1 and p21, are often over-expressed in many human cancers and their levels are correlated with high tumor grade, poor prognosis, and increased metastasis in subsets of carcinomas such as breast, prostate, cervical carcinomas and lymphomas [42,43]. We previously demonstrated that p21 is a transcriptional co-regulator of Smad that mediates TGFβ-induced breast cancer cell migration and invasion in metastatic breast cancer cells [44]. This prompted us to explore the roles of other cell cycle regulators in promoting tumor progression in breast cancer, aside from their well-established functions in cell cycle regulation. Thus, we investigated the effects of cyclins, in particular cyclin D1, downstream of TGFβ-mediated tumor progression. Indeed, several studies have supported the notion that the oncogenic effects of cyclin D1 may not be simply due to enhanced tumor cell growth or proliferation. These include reports showing a lack of correlation between cell proliferation and cyclin D1 expression in several large cohorts of 779 breast cancer patients [45,46] and the fact that elevated cyclin D1 expression is associated with a high incidence of metastasis and poor survival outcome [47,48], suggesting that cyclin D1 may play a role in promoting invasiveness of established tumors.

In this study, we found that TGFβ induced mRNA and protein expression of cyclin D1 in breast cancer cells with a highly migratory phenotype. Moreover, we found TGFβ to induce complex formation and nuclear co-localization of cyclin D1 and p21, indicating that these two proteins may cooperate to mediate TGFβ functions in aggressive human breast cancer cells. Furthermore, using gene silencing approaches, our results indicate that TGFβ-mediated cyclin D1 expression is a prerequisite for TGFβ-induced breast cancer cell migration. Orthotopic injection of cyclin D1/p21 null human breast cancer cells in nude mice considerably reduced mammary tumor growth *in vivo*, compared to animals injected with parental tumor cells. Moreover, we found that following fat pad transplantation, parental breast cancer cells invaded into the surrounding mammary tissues, while these effects were blocked when cyclin D1 and p21 gene expression were silenced. Collectively, these data indicate that TGFβ-mediated cyclin D1 and p21 gene expression leads to increased breast cancer migration and invasion *in vitro *and that blocking expression of these two cell cycle regulators in aggressive human breast tumors significantly reduced both tumor formation and local tumor invasion into the surrounding tissues *in vivo*.

## Methods

### Cell culture and transfection

Human breast cancer cell lines MDA-MB-231 (hereafter referred to as MDA) and SCP2 (provided by Dr. Joan Massagué) were cultured in DMEM containing 10% fetal bovine serum (FBS) and 2 mM L-glutamine. SUM149PT, SUM159PT and SUM229PE (provided by Dr. Stephen P. Ethier) were plated in F-12 HAM'S nutrient mixture (HyClone Laboratories, Inc., Logan, UT, USA) containing 5% FBS, 5 µg/ml insulin (Sigma-Aldrich, St. Louis, MO, USA), and 1 µg/ml hydrocortisone (Sigma). SUM1315MO_2 _were cultured in F-12 HAM'S nutrient mixture (HyClone) containing 5% FBS, 5 µg/ml insulin (Sigma), and 10 ng/ml epidermal growth factor (EGF) (Sigma). All cell lines were grown at 37°C in 5% CO_2_. Before stimulation with 5 ng/ml TGFβ1 (PeproTech, Rocky Hill, NJ, USA), cells were serum-starved overnight. For cell transfection, flag-tagged p21 cDNA (Addgene plasmid 16240), HA-tagged cyclin D1 cDNA (Addgene plasmid 11181), scrambled and cyclin D1 siRNAs (Sigma) were transfected using Lipofectamine™ 2000 (Invitrogen, Carlsbad, CA, USA), according to the manufacturers' protocols.

### Western blot analysis and immunoprecipitation

Protein extraction buffer containing 10 mM Tris-HCl, pH 7.5, 5 mM EDTA, 150 mM NaCl, 30 mM sodium pyrophosphate, 50 mM sodium fluoride, 1 mM sodium orthovanadate, 1% Triton X-100 and protease inhibitors (1 mM phenylmethylsulfonyl fluoride, 10 µg/ml leupeptin hydrochloride, 10 µg/ml aprotinin and 10 µg/ml pepstatin A) were freshly prepared and kept at 4°C before cell lysis. After cell lysates were centrifuged at 14,000 rpm for 15 minutes at 4°C, the concentration of total protein was quantified using a BCA protein assay kit (Thermo Scientific, Rockford, IL, USA). Cell lysates were boiled with 6× sodium dodecyl sulfate (SDS) Laemmli sample buffer for five minutes and subjected to immunoblot using mouse anti-p21 and rabbit anti-cyclin D1 antibodies (1:1,000 dilution, Santa Cruz Biotechnology, Santa Cruz, CA, USA). p21 and cyclin D1 were immunoprecipitated overnight at 4°C using their respective antibodies and followed by the addition of protein G-Sepharose beads (GE Healthcare Bio-Sciences, Piscataway, NJ, USA) for one hour at 4°C. The immunocomplexes were washed four times with cold lysis buffer and then subjected to Western blot.

### Real-Time PCR

TRIzol reagent (Invitrogen) was used to extract total RNA and reverse transcription of total RNA was carried out using M-MLV reverse transcriptase and random primers (Invitrogen) according to the manufacturer's instructions. SsoFast™EvaGreenÒ Supermix (Bio-Rad, Hercules, CA, USA) was used for amplification of the cyclin D1 mRNA in a Rotor Gene 6000 PCR detection system (MBI Lab Equipment, Montreal Biotech Inc., Kirkland, QC, Canada). The conditions for PCR were as follows: 95°C for 30 s, 40 cycles (95°C for 5 s and 59°C for 20 s). The primer sequences were as follows: cyclin D1 forward primer, AGCTGTGCATCTACACCGAC; reverse primer, ACTCCAGCAGGGCTTCGATCTG; *GAPDH *forward primer, GCCTCAAGATCATCAGCAATGCCT; reverse primer, TGTGGTCATGAGTCCTTCCACGAT.

### Kinetic cell migration assay

Cell migration was performed as previously described [44]. Briefly, 50,000 cells per well were cultured in Essen ImageLock 96-well plates (Essen Bioscience, Ann Arbor, MI, USA). The confluent cell layers were scratched to generate a wound using the Essen Wound maker. Cells were then treated in the presence or the absence of 5 ng/ml of TGFβ1. The images/videos of the wound were automatically taken at the exact same location using the IncuCyte™software (Essen Bioscience). Wound width, wound confluence or relative wound density were automatically measured by the IncuCyte software.

### Transwell cell migration assay

Transfected cell suspensions (40,000 cells/well) were seeded in 24-well Cell Culture Inserts (8-µm pore size; BD Biosciences, Mississauga, ON, Canada). After 24 hours incubation, the cells that migrated to the bottom of the membrane were fixed with 3.7% formaldehyde for 10 minutes and then labeled with the near-infrared fluorescence DNA binding dye DRAQ5 (2 µg/ml in PBS) at 37ºC for 5 minutes. The fluorescence intensity of migrated cells was measured at 700 nm in a near-infrared fluorescence imager (Odyssey CLX, LI-COR Biosciences - Biotechnology, Lincoln, NE, USA) using the Image Studio software (LI-COR Biosciences - Biotechnology).

### Immunofluorescence microscopy

For the invadopodia formation assay, cells were grown on top of eight-well chamber slides coated with 100 µl growth factor-reduced Matrigel. After TGFβ treatment for 24 hours, cells were fixed with 3.7% formaldehyde for 10 minutes, permeabilized in 0.1% Triton X-100 for 3 minutes, and blocked for 1 hour in 2% bovine serum albumin (BSA). Fixed cells were incubated with primary antibodies against p21, cyclin D1, F-actin and vimentin for one hour and followed by the secondary antibodies Alexa Fluor®568 goat anti-rabbit IgG and Alexa Fluor®488 goat anti-rabbit (1:800 dilution; Invitrogen) for one hour. Nuclei were stained with DAPI (Invitrogen). Confocal analysis was performed using a Zeiss LSM 510 Meta Axiovert confocal microscope (Carl Zeiss, Oberkochen, Baden-Württemberg, Germany) using the 63× objective.

### Mammary fat pad injection of nude mice

The animal study and SCP2 cells used in the mice model were approved by the McGill ethics committee (University Animal Care Committee, UACC) and all the experimental animal protocols were in accordance with the McGill University Animal Care. Four- to six-week old female Balb/c nude mice (Charles River Laboratories International, Wilmington, MA, USA) were used as a model for assessing mammary tumor formation and local invasion. An anesthetic cocktail of ketamine (50 mg/kg), xylazine (5 mg/kg) and acepromazine (1 mg/kg) was injected intramuscularly into mice (six per group). Fifty thousand parental SCP2 cells or p21 and cyclin D1 double knockdown SCP2 cells in 100 μl of saline (20% Matrigel) were injected into the mice mammary fat pads using a 30-gauge needle. Tumor growth and size were measured using a caliper. At eight weeks post-injection, mice were sacrificed and mammary tumors with surrounding skin and tissues were fixed in 10% neutral-buffered formalin for one day. Sections of mammary tumor were embedded in Tissue-Tek O.C.T. (VWR International, Radnor, PA, USA) compound and 9 µm thick sections were stained with hematoxylin and eosin to assess local advanced features, including skeletal muscle, mammary fat pad, and lymphovascular invasion as well as skin ulceration. Images of the tumors were photographed by light microscope using 10× and 20× objectives.

For intratibia injections, parental and p21/cyclin D1-depleted SCP2 cells (2.0× 10^6^) were injected intramuscularly into the left tibia of two group mice (six per group). The mice were monitored weekly for tumor burden. Digital radiography of hind limbs of all animals was used to monitor the development of skeletal lesions at four, six and eight weeks post-injection in a MX-20 cabinet X-ray system (Faxitron X-ray Corp.). On week 8, radiographs of anesthetized mice were taken.

### Statistical analyses

The difference between groups was analyzed using Student's *t*-test, and *P <0.05 was considered statistically significant.

## Results

### TGFβ induces cyclin D1 expression in highly migratory breast cancer cells

**Figure 1:**
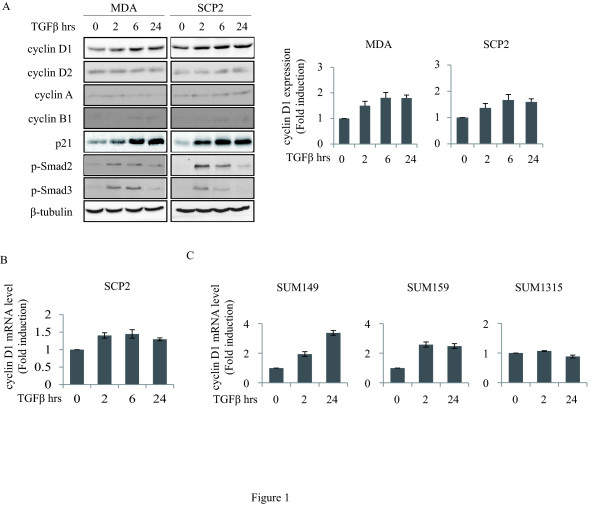
**TGFβ induces cyclin D1 expression in highly migratory breast cancer cells**. **(A) **MDA and SCP2 cells were stimulated with or without 5 ng/ml transforming growth factor beta (TGFβ) for the indicated times. Total cell lysates were subjected to immunoblot using cyclin D1, cyclin D2, cyclin A, cyclin B1, p21, p-Smad2, p-Smad3 and β-tubulin antibodies. Densitometry analysis of cyclin D1 protein expression was performed on four separate independent experiments (right panel). (**B and C) **cyclin D1 mRNA levels were measured by real-time PCR (error bars indicate SEM; *n *= 3 independent experiments) for the indicated cell lines.

We have previously shown that TGFβ's pro-migratory and invasive effects are mediated through the induction of p21 in highly migratory triple negative breast cancer cells [44]. Due to the fact that p21 is a universal regulator of cyclin/CDKs, this prompted us to investigate whether additional cell cycle regulators downstream of TGFβ are involved in this process. Moreover, because multiple studies have suggested that the oncogenic effects of cyclins may not be simply due to enhanced tumor cell growth or proliferation but may also involve tumor promoting functions [45-48], we examined the effect of TGFβ on protein expression levels of cyclins A, B1, D1 and D2 in the human aggressive breast cancer cell lines MDA and its metastatic sub-progeny SCP2 [49]. As shown in Figure 1A, we found that TGFβ significantly increased cyclin D1 protein expression in a time-dependent fashion. The effect of TGFβ on cyclin D1 expression was specific, as protein levels of G1 and S phase regulators cyclin D2 and A remained unchanged in response to TGFβ stimulation. The M phase cyclin B1 was barely detectable. As a positive control, we measured the expression of p21, which we have previously shown to be potently induced by TGFβ in MDA cells [44]. TGFβ induced the expression of p21 in a similar temporal expression pattern as cyclin D1 in these breast cancer cells. To assess whether TGFβ regulates cyclin D1 at the transcriptional level, we measured mRNA levels of cyclin D1 by quantitative PCR in SCP2 cells stimulated with TGFβ for 2, 6 and 24 hours. Induction of cyclin D1 mRNA by TGFβ was already detectable at 2 hours and was sustained for up to 24 hours (Figure 1B). These results highlight cyclin D1 as a novel TGFβ downstream target gene in human breast cancer cells.

To determine whether there was an association between TGFβ induction of cyclin D1 and TGFβ's pro-migratory effect, we measured the mRNA level of cyclin D1 in a panel of triple negative breast cancer cell lines which are either insensitive (SUM1315) or responsive (SUM149 and SUM159) to TGFβ-mediated cell migration and invasion [44]. Interestingly, TGFβ potently and persistently up-regulated cyclin D1 mRNA in the highly migratory cell lines SUM149 and SUM159, but not in the TGFβ-insensitive SUM1315 cell line (Figure 1C). Together, these results indicate that TGFβ-induced cyclin D1 expression correlates with TGFβ-induced p21 gene expression and cell migration, thus, suggesting that cyclin D1 may be associated with p21 and participate in TGFβ tumor-promoting functions in breast cancer cells.

### TGFβ promotes cyclin D1 nuclear accumulation and co-localization with p21

**Figure 2:**
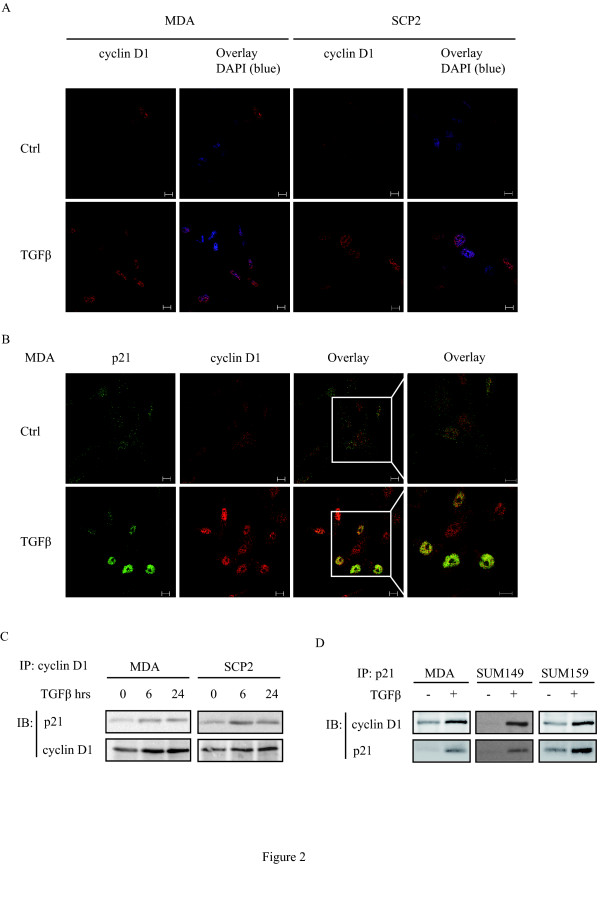
**TGFβ promotes cyclin D1 nuclear accumulation and co-localization with p21**. **(A) **MDA and SCP2 cells were treated with or without 5 ng/ml transforming growth factor beta (TGFβ) for 24 hours. Immunocytochemistry was performed using cyclin D1 (red) antibody and DAPI (blue). The scale bar is 10 µm. **(B) **MDA cells were immunostained using p21 (green) and cyclin D1 (red) antibodies. Co-localizaton (yellow) between p21 and cyclin D1 is represented by overlay. **(C) **MDA and SCP2 cells were treated with TGFβ for the indicated times. Cell lysates were analyzed by co-immunoprecipitation using an anti-cyclin D1 antibody. Immunoprecipitated cyclin D1 was subjected to Western blotting. **(D) **MDA, SUM149 and SUM159 cells were treated with TGFβ. Cell lysates were immunoprecipitated using an anti-p21 antibody and analyzed by immunoblotting using anti-cyclin D1 and anti-p21 antibodies.

The intracellular localization of cyclin D1 is important for its function and is, therefore, tightly regulated [50]. Constitutive accumulation of cyclin D1 in the nucleus has been shown to promote tumor transformation [51]. To determine whether TGFβ regulates cyclin D1 nuclear localization, we assessed the localization of cyclin D1 in MDA and SCP2 cells treated with or without TGFβ for 24 hours by confocal immunofluorescence microscopy. Cyclin D1 was predominantly found in the cytosol in unstimulated cells, whereas it appeared to be primarily retained within the nucleus after treatment with TGFβ (Figure 2A).

We have previously shown that TGFβ induces protein expression and nuclear localization of p21 in triple negative breast cancer cells [44]. The concurrent TGFβ effect on p21 and cyclin D1 prompted us to determine whether these molecules co-localize within the nucleus in response to TGFβ. As shown in Figure 2B, TGFβ facilitates nuclear co-localization of cyclin D1 and p21 in MDA cells. The simultaneous induction and co-localization in the nucleus of cyclin D1 and p21 by TGFβ suggested that they may be physically associated with each other. To address this, we performed co-immunoprecipitation of p21 and cyclin D1 in MDA and SCP2 cells treated with or without TGFβ for 6 or 24 hours. As shown in Figure 2C, TGFβ stimulated the interaction between endogenous p21 with cyclin D1 in a time-dependent fashion in MDA and SCP2 cells. Reciprocal immunoprecipitation experiments confirmed that endogenous cyclin D1 specifically interacts with immunoprecipitated p21 in response to TGFβ in MDA cells (Figure 2D, left panel). Moreover, the induction of complex formation between endogenous cyclin D1 and p21 was also observed in both SUM149 and SUM159 cells (Figure 2D, middle and right panels). Collectively, these results indicated that TGFβ stimulates the formation of a complex between cyclin D1 and p21 in triple negative basal-like breast cancer cells.

### Cyclin D1 is required for TGFβ-mediated cell migration

**Figure 3:**
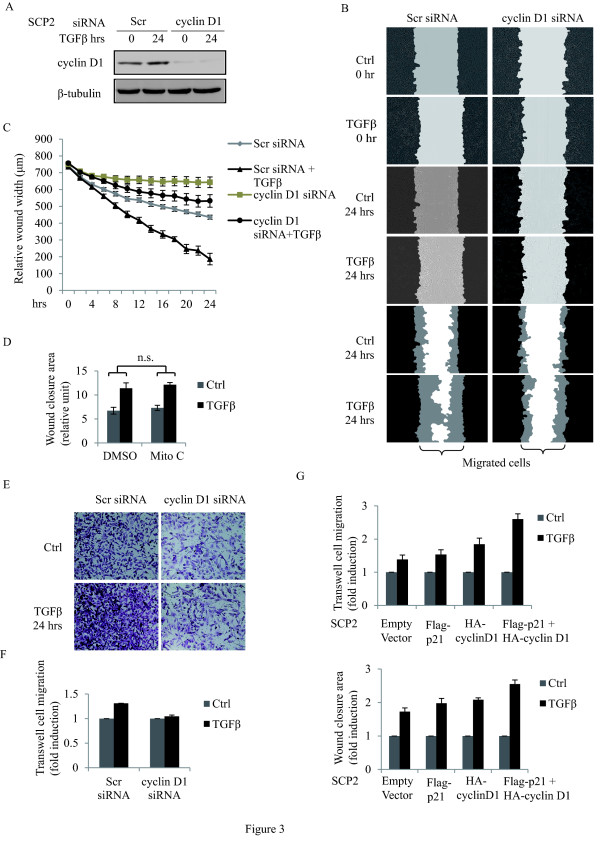
**Cyclin D1 is required for TGFβ-mediated cell migration**. **(A) **SCP2 cells were transfected with Scr or cyclin D1 siRNAs and then treated with or without 5 ng/ml transforming growth factor beta(TGFβ) for 24 hours. Total cell lysates were analyzed for cyclin D1 and β-tubulin by Western blotting. **(B) **Representative images of phase contrast and wound mask of transfected SCP2 cells stimulated without or with TGFβ for 0 and 24 hours in scratch/wound healing assay. The initial wound mask (black) and wound closure (grey) were measured using the Essen Instruments Scratch Wound Module. **(C) **Relative wound width was analyzed by the IncuCyte™software (Essen Bioscience) and quantified for the indicated times (error bars indicate SEM; *n *= 3 independent experiments). **(D) **SCP2 cells were treated with either vehicle (dimethyl sulfoxide, DMSO) or mitomycin C (Mito C) in the presence or absence of TGFβ. SCP2 cell migration was quantified using wound closure area at 24 hours (error bars indicate SEM; *n *= 3 independent experiments). **(E) **Representative images of transfected SCP2 cells stimulated with or without TGFβ for 0 and 24 hours in Transwell cell migration assay. Cells were stained with 0.2% crystal violet. **(F) **Transfected and migrated SCP2 cells in Transwell migration assay were stained with DRAQ5 fluorescent dye and quantified using fluorescent density at 24 hours (error bars indicate SEM; *n *= 3 independent experiments). **(G) **SCP2 cells were transfected with empty vector, Flag-p21, and HA-cyclin D1 separately or in combination. TGFβ-mediated cell migration was assessed using the Transwell (top panel) and wound healing (bottom panel) assays. Migration of the cells was quantified using fluorescent density (Transwell assay) and wound closure area (wound healing assay) at 24 hours (Error bars indicate SEM; *n *= 3 independent experiments).

Given that TGFβ enhanced cyclin D1 and p21 expression and complex formation in these human metastatic breast cancer cells, we investigated whether the TGFβ pro-migratory effect is mediated through cyclin D1. To address this, SCP2 cells were transfected with scrambled (Scr) siRNA or cyclin D1 siRNA. Cell migration in response to TGFβ was assessed by the scratch/wound healing assay coupled to quantitative time-lapsed imaging for up to 24 hours. As shown in Figure 3A, TGFβ-induced cyclin D1 protein expression in the SCP2 cells transfected with Scr siRNA was blocked in cells transfected with cyclin D1 siRNA. As shown in Figure 3B, C, while TGFβ stimulated rapid wound closure in SCP2 cells transfected with the Scr siRNA, this effect was delayed in SCP2 cells depleted of cyclin D1. TGFβ-induced wound closure was not affected by the mitotic inhibitor mitomycin C, suggesting that the effect of TGFβ on cell migration was independent of cell proliferation (Figure 3D). We further assessed the role of cyclin D1 downstream of TGFβ-mediated cell migration, using a Transwell migration assay. As shown in Figure 3E, F, knocking down cyclin D1 inhibited the TGFβ pro-migratory effects, consistent with what observed with the wound healing assay (Figure 3B, C).

To then address whether cyclin D1 and p21 have any synergistic effect, p21 and cyclin D1 cDNAs were overexpressed alone or in combination and the TGFβ effect on cell migration was examined using both the wound healing and Transwell migration assays. As shown in Figure 3G, overexpression of cyclin D1 or p21 alone had little or no potentiation effect on TGFβ-induced cell migration. However, overexpression of both proteins clearly increased/potentiated the TGFβ effect, suggesting that these two proteins synergize their effect downstream of TGFβ. This is consistent with our main finding and conclusion, showing that the two proteins cooperate to regulate TGFβ-mediated breast cancer cell migration and tumor local invasion. Together, these results demonstrate that cyclin D1 is required for TGFβ-mediated migration in breast cancer cells.

### Cyclin D1 is a downstream mediator in TGFβ-regulated actin reorganization and invadopodia formation

**Figure 4:**
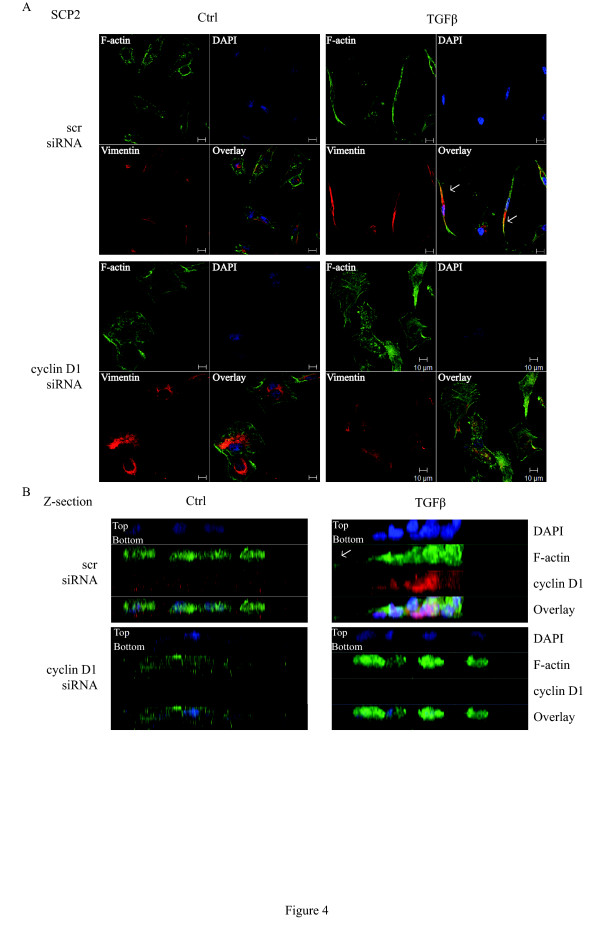
**Cyclin D1 is a downstream mediator in TGFβ-regulated actin reorganization and invadopodia formation**. **(A) **Scr siRNA- or cyclin D1 siRNA-transfected SCP2 cells were treated with or without 5 ng/ml transforming growth factor beta (TGFβ) for 24 hours. Immunocytochemistry was performed using F-actin (green) antibody, vimentin (red) antibody and DAPI (blue). Co-localizaton (yellow) between F-actin and vimentin is represented by overlay. The scale bar is 10 µm. **(B) **Scr siRNA- or cyclin D1 siRNA-transfected SCP2 cells were cultured on the top of growth factor-reduced Matrigel and then treated with or without 5 ng/ml TGFβ for 24 hours. Immunocytochemistry was performed using F-actin (green) and cyclin D1 (red) antibodies and DAPI (blue).

Cyclin D1 has previously been reported to regulate cellular migration in primary bone macrophages, mouse embryo fibroblasts (MEFs), and breast cancer cells [52-54]. For instance, cyclin D1-deficient MEFs display a more spread phenotype, and an increased cell adhesion and actin stress fiber formation through inhibition of thrombospondin 1 and ROCK signaling [53]. Therefore, we examined whether cyclin D1 effects on cellular structure and actin organization contribute to TGFβ-mediated cancer cell migration. To this end, SCP2 cells transfected with either Scr or cyclin D1 siRNAs were stimulated with TGFβ and the dynamics of actin organization were assessed by staining with the fluorescently labeled F-actin marker phalloidin and mesenchymal intermediate filament vimentin. As shown in Figure 4A, vimentin filaments co-localized with F-actin at the leading edge of aggressive SCP2 cells transfected with Scr siRNA, which displayed an elongated phenotype in response to TGFβ. Interestingly, cyclin D1-deficient cells were rounded and exhibited more epithelial-like phenotype. Furthermore, suppression of cyclin D1 expression not only prevented the elongation of vimentin filaments, but also the co-localization with F-actin at the cell edge.

Vimentin is required for the elongation of invadopodia subcellular structures, which are three-dimensional actin-rich protrusions [55]. Invadopodia are selectively found in invasive cancer cells and are important for the degradation of the ECM [56]. As cyclin D1 affects vimentin distribution, we investigated whether cyclin D1 could regulate invadopodia formation. SCP2 cells transfected with either Scr or cyclin D1 siRNAs were seeded on top of growth factor-reduced Matrigel and treated with or without TGFβ. Whereas Scr-transfected SCP2 cells stimulated with TGFβ showed increased F-actin-bundled protrusion and invaded into the Matrigel, this phenotype was completely abolished by knocking down cyclin D1 expression (Figure 4B). All together, these results defined novel functions for cyclin D1 as a TGFβ downstream target that is required for this growth factor to mediate vimentin elongation, induction of a migratory morphological phenotype, and the formation of invasive subcellular structures in metastatic breast cancer cells.

### Depletion of cyclin D1 and p21 prevents mammary tumor growth and local invasion

**Figure 5:**
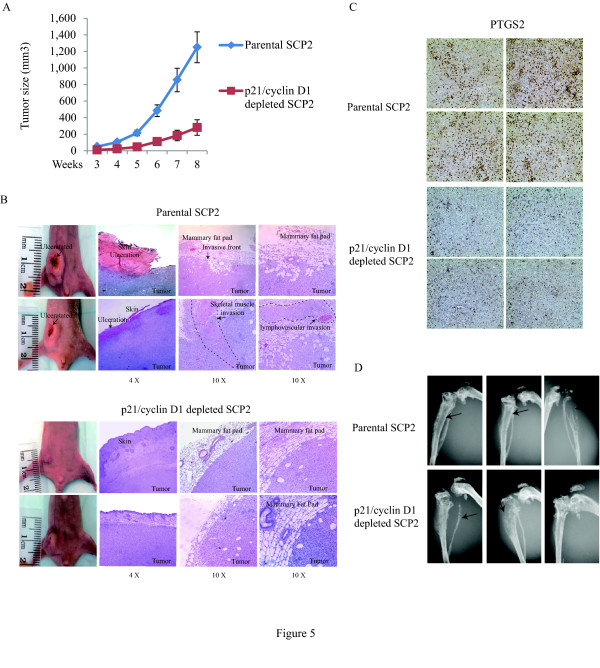
**Depletion of cyclin D1 and p21 prevents mammary tumor growth and local invasion**. **(A) **Parental SCP2 and p21/cyclin D1 double knockdown SCP2 cells were implanted into the mammary fat pad of four- to six-week-old female Balb/c nude mice. Mammary tumor growth was measured from two sets of mice and quantified for the tumor size at the indicated times (six per group; error bars indicate SEM). **(B) **Representative photographs show hematoxylin and eosin staining of the mammary gland (tumor and surrounding tissues) of mice at eight weeks post-injection. **(C) **Representative photographs show PTGS2 staining of parental and p21/cyclin D1-depleted mammary tumor of mice at eight weeks post-injection. **(D) **Representative radiographs of skeletal lesions in two groups of mice (parental and p21/cyclin D1-depleted SCP2) were taken by X-ray using Faxitron. Parental and p21/cyclin D1-depleted SCP2 cells were injected in tibia. The lesions are highlighted by arrows.

Overexpression of p21 and cyclin D1 is correlated with poor prognosis and aggressiveness in breast cancer. To address the importance of p21 and cyclin D1 on breast cancer development *in vivo*, we injected either SCP2 control or double p21 and cyclin D1 knockdown cells into the mammary fat pads of female Balb/c nude mice to monitor primary tumor growth and local invasiveness. Silencing p21 and cyclin D1 expression using siRNAs significantly reduced the rate of primary tumor formation and tumor size (Figure 5A). As depletion of p21 alone did not affect tumor formation in a Xenograft transplantation *in vivo *model [55], it is likely that the observed phenotype on tumor formation in the double knockdown is mediated by cyclin D1. This is in agreement with previous studies showing that depletion of cyclin D1 prevented tumor development in oncogenic HER2 overexpressing transgenic mice [57-59]. Importantly, three out of six mice in the control group had tumors ulcerating through the overlaying skin, while all the mice in the double knockdown group had intact skin. Breast tumor with ulcerated skin has been clinically classified as locally advanced breast cancer. All tumors were taken with the overlaying skin and surrounding tissues and subjected to hematoxylin and eosin staining. As shown in Figure 5B, the deep tumor margins in the control group were less distinct, invading nearby structures, including skeletal muscles and the mammary fat pad, and showed frequent lymphovascular invasion. However, the tumor margins in the knockdown group were well encapsulated with a non-invasive nature. In addition, we performed immunohistochemistry on primary mammary tumor derived from animals injected with parental and p21/cyclin D1-depleted SCP2 cells. We assessed the expression of the TGFβ-regulated gene PTGS2, which we have previously shown to be involved in mediating the TGFβ effect on cell migration and invasion [44]. As shown in Figure 5C, using tumors from four different mice in each group, we found expression of PTGS2 to be clearly higher in parental tumors compared to p21/cyclin D1-depleted tumors, further confirming that the p21/cyclin D1-depleted tumors displayed less invasive features.

To investigate the role of p21 and cyclin D1 on the development of bone osteolytic lesions, parental and double knockdown SCP2 cells were injected intramuscularly into the left tibia of two groups of nude mice. As shown in Figure 5D, following X-ray examination of the bones, both group of mice developed secondary tumors that caused severe osteolytic bone lesions, suggesting that p21/cyclin D1 do not affect the later stages of bone metastasis. Collectively, these results indicate that while p21 and cyclin D1 are required for breast cancer cells to acquire an invasive phenotype, their effects are primarily occurring at the earlier stages of tumor metastasis, namely induction of local cell invasion from the tumor to the surrounding tissues. This is also consistent with previous work, showing that depletion of p21 alone did not affect the development of bone osteolytic lesions [44].

## Discussion

Cyclin D1 is a well-characterized oncogene that is frequently overexpressed in human breast, lung, colon, prostate and hematopoietic carcinomas [60-62]. This is a unique feature among the three closely related D type G1 cyclins (D1, D2 and D3), as amplification of cyclin D2 and D3 copy-number is rarely observed in human cancer. In fact, methylation of cyclin D2 resulting in loss of its expression has been reported in breast, pancreatic and prostate cancer [63-65]. In addition to the association between cyclin D1 expression and human cancer, overexpression of cyclin D1 is tumorigenic, as supported by evidence that MMTV-driven cyclin D1 is sufficient for mammary hyperplasia and carcinoma development in transgenic mice [66]. Furthermore, cyclin D1 is required for many oncogenes, such as HER2 or Ras, to induce mammary tumor growth in mice [57-59]. The function of cyclin D1 in mammary oncogenesis in mice is mediated through the activation of its regulatory partner CDK4, as mice lacking CDK4 or expressing the CDK4/CDK6-specific inhibitor INK4A are resistant to HER2-induced mammary tumor formation [58,67-69]. While these studies addressed the importance of cyclin D1 on breast tumor initiation, its contribution to the development and progression of established tumors remains unclear.

Several studies support the notion that the oncogenic effects of cyclin D1 may not be simply due to enhanced tumor cell growth or proliferation. For instance, cyclin D1 expression did not correlate with Ki67 expression in a cohort of 779 breast cancer patients [45]. In another study of 1,740 breast cancer patients, cyclin D1 expression was not tightly associated with proliferative genes that are regulated by the inactivation of CDK4 substrate RB [46]. In addition, high expression of cyclin D1 is associated with high incidence of metastasis and poor survival outcome [47,48]. Therefore, cyclin D1 is potentially required for continual development and progression of established tumors.

In this study, we investigated the function of cyclin D1 on breast tumor progression induced by TGFβ, a potent tumor-promoting factor, in metastatic breast cancer cell lines. Our results showed that the effect of TGFβ on cyclin D1 expression was specific, as protein levels of other cyclins in G1, S and M phase are unresponsive to TGFβ stimulation. Furthermore, using a panel of tumorigenic triple negative breast cancer cell lines, which exhibit differential responses to TGFβ in terms of cellular migration, we found cyclin D1 expression to correlate with p21 expression and to be required for TGFβ-induced cell migration. Furthermore, up-regulation of the cyclin D1 gene by TGFβ is more potent and persistent in highly migratory cell lines compared with less motile cells. This is consistent with a previous study using intravital imaging of live tumor-bearing nude mice, showing that although TGFβ signaling promotes single tumor cell migration and metastatic spread into blood vessels and lymph nodes, not all cells with active TGFβ signaling are migratory [12]. Our results suggest that cyclin D1 is a specific downstream target for TGFβ-mediated cell migration.

Subcellular localization and stabilization of cyclin D1 play an important role in human cancers [70]. We showed a TGFβ-induced nuclear localization of cyclin D1 in these metastatic breast cancer cell lines. It has been demonstrated that oncogenic actions of cyclin D1 are predominantly nuclear in many cancers, as carcinogenic mutations and deletions often occur at the T286 site, which controls cyclin D1 protein turnover and nuclear export [71,72]. Mutated cyclin D1 with constitutive nuclear localization and impaired degradation not only enhanced cyclin D1 transformation efficiency *in vitro*, but also promoted tumor formation *in vivo *[73]. Our study further revealed that TGFβ-induced nuclear cyclin D1 promotes cell migration by altering cell morphology and the formation of invasive subcellular structures in metastatic breast cancer cells.

Cyclin D1 has been recognized as a multifunctional protein, which regulates angiogenesis, lipogenesis, mitochondrial function and cell migration [53,54,74-78]. A recent study identified that more than 100 cyclin D1-interacting proteins are involved in the regulation of cell cycle, transcription, DNA repair, RNA metabolism, protein folding and cell structure [79], suggesting that these interactors might influence various biological functions of cyclin D1. It has been shown that p21 interacts with cyclin D1 to promote nuclear accumulation of cyclin D1 [80]. In addition, cyclin D1 associates with p21 to facilitate DNA repair, and this function of cyclin D1 is independent of CDK4 activation [81,82]. We demonstrated that in the context of TGFβ signaling, cyclin D1 associates with p21 in metastatic breast cancer cells. Furthermore, depletion of cyclin D1 and p21 prevented mammary tumor formation and subsequent local invasion into surrounding tissues. Our previous study showed that p21 is required for TGFβ-mediated cell migration and invasion; therefore, these results not only highlight cyclin D1 as a novel TGFβ downstream target, but also indicate that cyclin D1 cooperates with p21 to mediate the effect of TGFβ on breast cancer progression.

## Conclusions

In this study, we showed that TGFβ significantly induced cyclin D1 expression in metastatic breast cancer cells. TGFβ-induced cyclin D1 and p21 proteins remain mostly co-localized in the nucleus and physically interact with each other. Importantly, we found that up-regulated p21 and cyclin D1 play an important role in TGFβ regulation of cellular migration and invasion by actin remodeling. These results suggest that cyclin D1 and p21 may cooperate with each other to mediate the tumor-promoting effects of TGFβ in aggressive breast cancer cells.

## Abbreviations

BSA: bovine serum albumin; CDKs: cyclin-dependent kinases; CDKIs: cyclin-dependent kinases inhibitors; DMEM: Dulbecco's Modified Eagle Medium; DMSO: dimethyl sulfoxide; ECM: extracellular matrix; EGF: epidermal growth factor; EMT: epithelial to mesenchymal transition; FBS: fetal bovine serum; MEF: mouse embryo fibroblast; p21: p21CIP1/WAF1; R-Smads: receptor-regulated Smads; SDS: sodium dodecyl sulfate; TGFβ: transforming growth factor beta

## Competing interests

The authors declare that they have no competing interests.

## Authors' contributions

MD and JJL were involved in designing all experiments, and analyzing and interpreting data. MD performed the experiments and wrote the manuscript. JG was involved in the immunofluoresent experiment. NFA was involved in the cell migration experiment. MAV participated in the immunohistochemistry experiment. JJL, NFA, MAV and SA assisted in drafting and editing the manuscript. SA was involved in the study design and result analysis and interpretation. AAA analyzed tumor local invasiveness. SAR and AA performed *in vivo *studies and analyzed the mammary tumor growth. All authors read and approved the final manuscript.
