# Erratum to: Cyclin D1 cooperates with p21 to regulate TGFβ-mediated breast cancer cell migration and tumor local invasion

**DOI:** 10.1186/s13058-017-0831-8

**Published:** 2017-03-28

**Authors:** Meiou Dai, Amal A. Al-Odaini, Nadège Fils-Aimé, Manuel A. Villatoro, Jimin Guo, Ani Arakelian, Shafaat A. Rabbani, Suhad Ali, Jean Jacques Lebrun

**Affiliations:** 1 Division of Medical Oncology, Department of Medicine, McGill University Health Center, Royal Victoria Hospital, Montreal, QC Canada; 2 University of Dammam, Ministry of Higher Education, Riyadh, Saudi Arabia; 3 Department of Medicine, McGill University Health Center, Royal Victoria Hospital, Montreal, QC Canada

## Erratum

**Main text:** After publication of this work [1] an error was noticed in Fig. 5c. Figure 5c shows representative images/fields of PTGS2 staining of mammary tumors derived from mice injected with parental SCP2 cells versus mice injected with p21/cyclinD1 depleted SCP2 cells. Tissue slides from the two groups of animals were stained with an anti-PTGS2 antibody. Results showed a decrease in PTGS2 staining in the cyclinD1/p21 depleted group compared to the parental group.

**Fig. 5:**
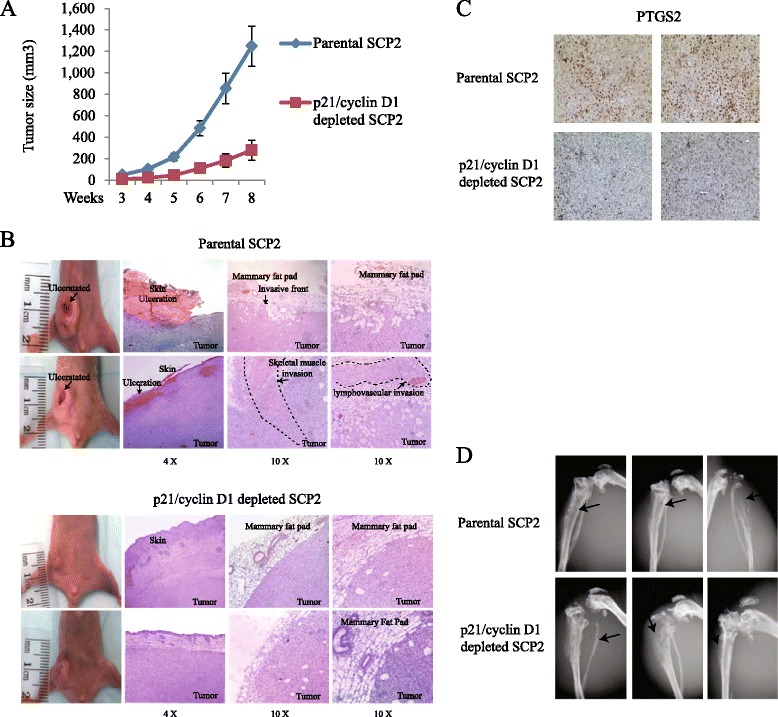
Depletion of cyclin D1 and p21 prevents mammary tumor growth and local invasion. **a** Parental SCP2 and p21/cyclin D1 double knockdown SCP2 cells were implanted into the mammary fat pad of four- to six-week-old female Balb/c nude mice. Mammary tumor growth was measured from two sets of mice and quantified for the tumor size at the indicated times (six per group; error bars indicate SEM). **b** Representative photographs show hematoxylin and eosin staining of the mammary gland (tumor and surrounding tissues) of mice at eight weeks post-injection. **c** Representative photographs show PTGS2 staining of parental (top panel) and p21/cyclin D1-depleted (lower panel) mammary tumors at eight weeks post-injection. Two mice were used in each group. **d** Representative radiographs of skeletal lesions in two group mice (parental and p21/cyclin D1-depleted SCP2) were taken by X-ray using Faxitron. Parental and p21/cyclin D1-depleted SCP2 cells were injected in tibia. The lesions are highlighted by arrows

The tumor images in Fig. 5c came from two different mice for each group and not 4 as mistakenly indicated. Two representative images were used for each mouse but were largely overlapping. Thus, to avoid confusion, we have removed the overlapping images and retained 1 representative image for each of the 4 mice (2 mice injected with parental SCP2 cells and 2 mice injected with p21/cyclinD1 depleted SCP2 cells). We apologize for this error, which did not affect any of the interpretations or conclusions of the article.
